# The Newcastle Eye Infirmary

**Published:** 1893-07-01

**Authors:** 


### The Institutional Workshop.

### WITHIN THE HOSPITALS.

### THE NEWCASTLE EYE INFIRMARY.

(By Our Special Commissioner.)

This is a new institution, founded seventy years ago, and now occupying new premises specially erected for the accommodation of the patients. Structurally the hospital, which contains twenty beds, leaves little to be desired, as the wards, though small (six beds), are well proportioned, and the lavatories and other accommodation satisfactory. The operation theatre is excellently lighted, but very small. There seems to be considerable pressure upon the bed space, as most of the wards were overcrowded; that is to say, they contained more beds than the available superficial and cubic space seemed to us to warrant. There is a well proportioned convalescent room, and comfortable out-patient department, with all necessary appliances and fittings for the attendance of a considerable number of patients. The hospital is in charge of a Matron, Miss Crump, who is warmly interested in the work, and to whose devotion to duty the present efficiency of the institution is in a large measure due. One of the honorary surgeons, Dr. Jeaffreson, fills the office of Honorary House Governor. This is a new departure, seeing that we know no institution where the post of House Governor has heretofore been held by a medical man, or where so small a hospital has found it necessary to have the services of so exalted an official. The hospital is free, there being no letters of recommendation. A small charge of from 3d. to 6d. is made for medicine, except in cases of extreme poverty. In-patients who are destitute may be admitted free to a limited extent, but a large majority pay from 1s. to 2s. per diem, it being usual for patients to bring a guarantee with them securing this payment, signed by some well-known charitable person in the neighbourhood where they reside. The amount received from patients' payments in 1891 was, in round figures, £142, of which in-patients paid about £23. Patients are retained on an average about ten days, so that, taking the lower rate of 1s. per patient, out of the whole number of 317 patients last year half paid nothing for their treatment, despite the resolution to the contrary. We should like to hear an explanation on this point, seeing that the patients' contributions bear an important relation to the well-being and success of special hospitals. Cases appear to be sent from all over the counties of Northumberland and Durham, and from even greater distances. We would call the Honorary House Governor's attention to a curious mistake on page six of the report, which is headed "Imfluenza and Eyesight." The report states that it is desirable, in the interests of the miners, to add a special ward at once for the treatment and reception of cases of suppurative keratitis. At present, owing to the want of accommodation, the failure on the part of the miners to apply at once for treatment results in many of them losing their eyesight in from one to three days after accidents, whereas immediate admission to the hospital would be the means of saving many eyes which are now irretrievably lost. The hospital is wanted in Newcastle for the protection as well of the public, and we commend the suggestion of the report to the favourable consideration of those who are able to contribute to a special fund.

